# Perceptions, needs and preferences of chronic disease self‐management support among men experiencing homelessness in Montreal

**DOI:** 10.1111/hex.13106

**Published:** 2020-09-23

**Authors:** Laura Merdsoy, Sylvie Lambert, Jessica Sherman

**Affiliations:** ^1^ Ingram School of Nursing McGill University Montreal QC Canada; ^2^ St. Mary's Research Centre Montreal QC Canada; ^3^ Welcome Hall Mission Montreal QC Canada

**Keywords:** homeless, chronic disease, self‐management, qualitative, vulnerable population, men's health

## Abstract

**Objective:**

This study explored the perceptions, needs and preferences for chronic disease self‐ management (SM) and SM support among men experiencing homelessness.

**Design:**

A qualitative interpretive approach was used. Eighteen semi‐structured interviews were conducted with 18 homeless men with a chronic disease at an emergency overnight shelter of Welcome Hall Mission (WHM) in Montreal, Quebec. Interviews were audio‐recorded, transcribed verbatim and thematically analysed.

**Results:**

The majority of participants perceived SM as important, described confidence to perform medical SM behaviours, and creatively adapted their SM behaviours to homelessness. Emotional SM was described as most challenging, as it was intertwined with the experience of homelessness. Three vulnerable groups were identified: (a) those with no social networks, (b) severe physical symptoms and/or (c) co‐morbid mental illness. The preferred mode of delivery for SM support was through consistent contacts with health‐care providers (HCPs) and peer‐support initiatives.

**Discussion and Conclusions:**

Despite competing demands to fulfill basic needs, participants valued chronic disease SM and SM support. However, SM support must address complex challenges relating to homelessness including emotional SM, multiple vulnerabilities and barriers to forming relationships with HCPs.

## 1 INTRODUCTION

Characterized by the World Health Organization (WHO) as an invisible global epidemic, chronic disease is a leading cause of mortality worldwide.^1^ In addition to premature loss of life, a chronic disease diagnosis is associated with adverse health outcomes including reduced quality of life, increased emotional distress, loss of productivity and increased health‐care costs.^2^ A substantial burden to the Canadian health‐care system, 51.6 % of Canadians have at least one chronic disease, and 14.8% have two or more.^3^

Populations with low socio‐economic status (SES) have higher rates of chronic disease due to limited access to protective factors like nutritious food, safe housing, secure employment and health services.^4^ Most at risk among low SES populations are those experiencing homelessness, with as many as 85% reporting a diagnosis of chronic disease.^5^ Among this population, risks for developing a chronic physical disease include a higher prevalence of substance abuse, mental illness, exposure to the elements, poor sleeping and eating conditions, increased exposure to violence, and social stigma.^6^, ^7^

Self‐management (SM) is one of the six components of the Chronic Disease Model,^8^ and ‘relates to the tasks that an individual must undertake to live well with one or more chronic conditions’.^9^ Hence, SM is not only a series of tasks, but includes the notion of ‘confidence’. SM tasks can be categorized as medical management (eg monitoring symptoms, managing side effects), role management (eg maintain activities of daily living, creating a community of peers) and emotional management (eg dealing with shock, making sense of illness).^9^, ^10^, ^11^ Key patient SM behaviours in carrying out these tasks include the following: problem‐solving, action planning, decision‐making, locating and using resources, forming partnerships with HCPs and self‐monitoring.^12^ SM support is ‘the systematic provision of education and supportive interventions to increase patients' skills and confidence in managing their health problems, including regular assessment of progress and problems, goal setting and problem‐solving support’.^13^ SM support involves the collaboration between individuals with chronic diseases and a multidisciplinary team of health‐care providers through structured programmes and interventions to develop techniques and capacities to improve the management of chronic diseases.^14^ SM support is an effective strategy for improving disease outcomes, including appropriate use of health‐care services, medication compliance, symptom control, quality of life, self‐efficacy and motivation.^15^, ^16^, ^17^, ^18^, ^19^ Yet, despite evidence of the efficacy of SM support, research among low SES populations remains relatively scant. Scarcer still is research on chronic disease SM among populations experiencing homelessness.

A search of the literature by the authors yielded only three non‐randomized, pilot or feasibility intervention studies examining chronic disease SM among populations experiencing homelessness.^20^, ^21^, ^22^ The one‐group, pre‐post‐test pilot study by Hulton et al^20^ examined the feasibility of implementing the Stanford Chronic Disease Self‐Management Program (CDSMP)^23^ combined with nursing case management among ten sheltered homeless adults with a chronic illness. Six out of the ten participants completed the programme, with positive changes in health outcomes noted (notwithstanding the small sample size). The feasibility study by Henwood et al^21^ also explored the acceptability and feasibility of the CDSMP^23^ for health promotion and this among 15 men experiencing homelessness. All participants were retained, which can potentially be attributed to the highly participative study design. Qualitative interviews with participants revealed that the programme was helpful in learning the skills needed to address health concerns and feeling more empowered to do so. Also, peer‐support and participatory elements emerged as central components in ensuring the intervention's success. Last, the pilot study by Savage et al^22^ to assess retention of adults with diabetes and experiencing homelessness in a 12‐week SM support intervention (focused on managing diabetes and obtaining needed recourses) combined with nursing case management found that of the three participants assigned to the intervention, two completed the intervention. A pre‐post survey revealed improvement in some outcomes (eg cognitive symptom management); however, the very small sample size precludes any definitive conclusion.

Whereas the outcomes of these three studies are promising, their challenges with recruitment and retention may be partly explained by the dearth of exploratory research around the appropriateness of SM and SM support interventions among homeless populations. Furthermore, there is mixed evidence of the efficacy of adapting evidence‐based SM interventions to divergent contexts, such as that of chronic disease SM interventions among populations experiencing homelessness.^24^ Thus, further exploration of the basic acceptability and experience of SM support among this population is warranted. This study aimed to fill this knowledge gap by answering the question: what are the perceptions, needs and preferences of chronic disease SM support among men experiencing homelessness? Perceptions are defined as the ways in which something is regarded, understood or interpreted.^25^ Needs are defined as a lack of something requisite, desirable or necessary for avoiding harm and can be revealed through barriers and challenges to accessing services.^26^, ^27^ Preferences are defined, in the context of this study, as that which is valued as the ideal choice in regards to health‐care goods, services or interventions received.^28^ Given these parameters, the objectives of this study were to explore: (a) the perceived importance of SM and SM support among participants, (b) the barriers and challenges participants experience in developing SM behaviours or receiving SM support and (c) participants' opinions about ideal SM support services and programmes.

## 2 METHODS

Ethics approval for the study was obtained from the McGill University Faculty of Medicine Institutional Review Board and the shelter where this study took place.

### 2.1 Study design and setting

This study used a qualitative interpretive approach.^29^ No other methodological orientation underpinned the study. Interviews were conducted between August and December 2016 at the emergency overnight shelter of a non‐profit organization^30^ offering a variety of programmes and services to Montrealers living in poverty and social exclusion. This overnight shelter provides an evening meal, bathing and sleeping facilities for up to 222 men experiencing homelessness per night.

### 2.2 Sample and sample size

This study sought to recruit at least 12 participants, as this sample size has been found to often be sufficient in achieving data saturation in qualitative studies.^31^ Inclusion criteria for participants consisted of the following: (a) men over the age of 18 accessing the overnight shelter; (b) who self‐identified as living with a chronic physical disease, broadly defined as a non‐communicable disease diagnosed for at least one year; (c) and spoke English or French well enough to participate in the study; and (d) had no known history of violence or inappropriate behaviour. There were no restrictions based on time spent homeless, type of chronic disease, or physical or mental co‐morbidities. As a starting point, individuals' chronic physical diseases were categorized according to the types suggested by the Public Health Agency of Canada (A‐Z Chronic Diseases). However, given our broad definition, we accepted what participants' identified as the chronic disease they were coping with, which included more than the typical chronic physical diseases.

### 2.3 Recruitment procedures

Recruitment from the overnight shelter was coordinated by an intervention worker who identified which men were potentially eligible and then approached them (face‐to‐face) to provide a brief introduction to the study. Recruitment took place on nights where this was feasible for the intervention worker, given competing priorities. Individuals who agreed to participate were invited to meet with a Master's nursing student (first author) or a Research Assistant (RA) in a private room at the shelter, at which point more information about the study was provided. If participants were still interested in participating, the consent form was reviewed and participants were given time to ask questions before signing the consent form and commencing the interview. All participants were offered a choice between $5‐10 gift certificates to a local coffee shop or grocery vendor.

### 2.4 Data collection

Face‐to‐face, semi‐structured interviews were conducted by the first author (female master's student in nursing) with each participant in either English or French. This was the first author's qualitative study. There were no a priori relationships between the interviewer and participants (participants did not know anything about the interviewer before the interview). Interviews took between 45 and 90 minutes, including a sociodemographic questionnaire administered at the end of each interview. All interviews took place at the overnight shelter between the ‘check‐in’ hours of 15:30 and 18:00 and all (with the exception of one) were audio‐recorded. Field notes were kept to record the interviewers' impression of the interviews. A semi‐structured interview guide consisting of open‐ended questions was developed based on Lorig and Holman's SM framework.^12^ Examples of interview questions are: What is involved in managing your illness or illnesses? What kinds of things do you have to do? How do you cope emotionally with your chronic illness? Where and how have you received education or information about your chronic illness? There were no repeat interviews.

### 2.5 Data analysis

Data collection and analysis were conducted concurrently. All audio‐recordings were transcribed verbatim and transcriptions were verified against original audio‐recordings. Inductive thematic analysis was used as a method for identifying, analysing, and reporting patterns within the interview data through a process of coding.^32^ The 1st author coded all the transcripts, and codes were discussed at regular meeting with the 2nd and 3rd author.

**FIGURE 1:**
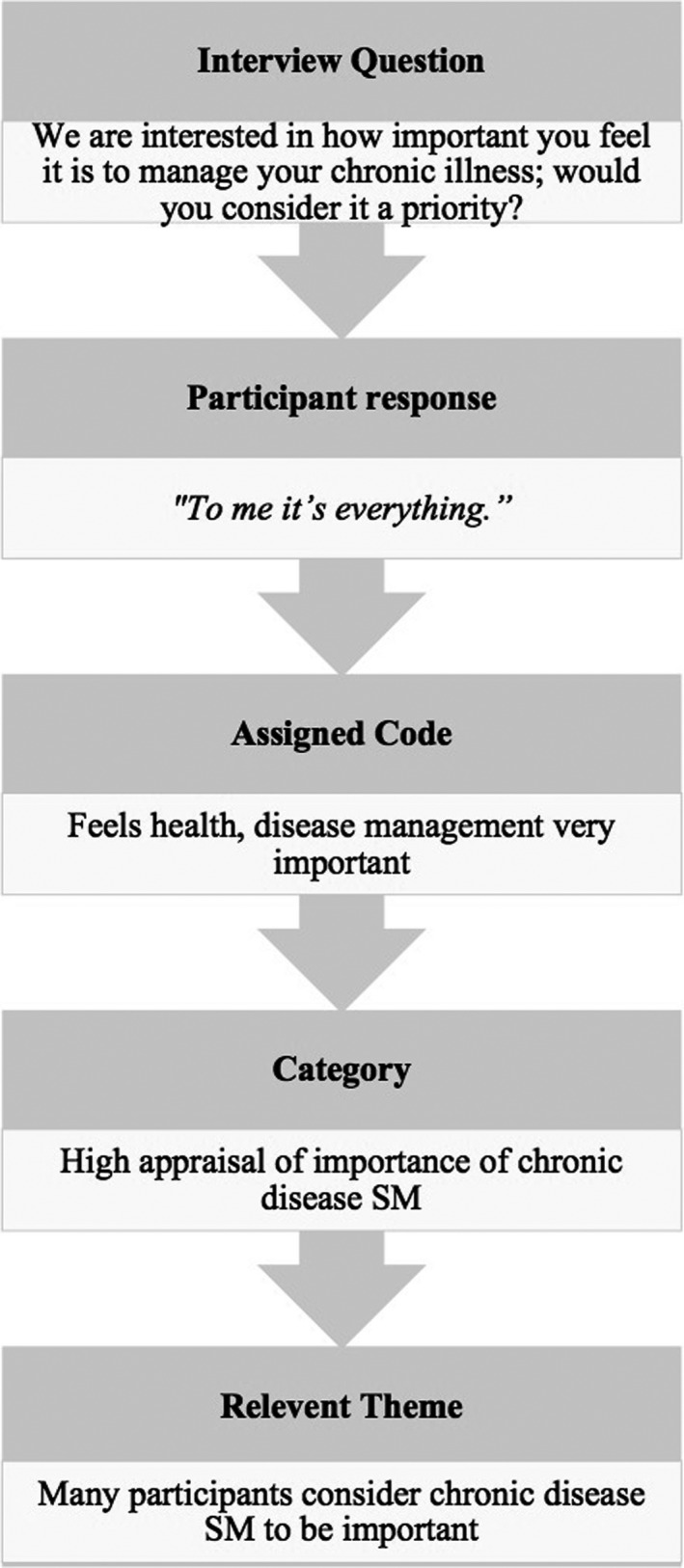
Example of coding process

Codes, the most basic segment of raw data that can be assessed in a meaningful way regarding a phenomenon,^32^ were identified by reading and re‐reading transcripts and applying short sentences to describe the data extracted. Codes were then organized into categories according to their shared similarities. Themes were derived from the data by analysing patterns that emerged.^32^ Transcripts were then re‐reviewed to refine and clarify themes, ensuring coherence across interviews and generating clear definitions.^33^ Transcripts were not returned to participants for comment and findings were not reviewed by participants. Figure 1 provides an example of the data analysis process. A summary of strategies used to ensure study rigour is available in Table 1. Microsoft Office Word and Transcribe were used as assistive software for data analysis and management.

**TABLE 1:** Evaluation of rigour in qualitative research^a^

Criteria	Explanation	Application during study
Credibility	Refers to the truth‐value of the data representing participants' experiences	Used member‐checking, paraphrasing, summarizing and/or repeating information during the interview to ensure that researcher interpretations of the data were consistent with participant statements Regularly de‐briefed among authors to review and validate emerging findings and recognize potential biases in data interpretation
Confirmability	Refers to how well the findings reflect the information provided by participants	Used an audit trail to document study context, participant characteristics, study procedures, decision‐making processes and the data analysis process
Transferability	Refers to the degree to which the results of qualitative research can be generalized to other contexts or settings	Documented thorough descriptions of the study setting and participants in reflexive field notes

^a^ Adapted from Thomas & Magilvy, 2011

## 3 RESULTS

### 3.1 Sample description

The number of people approached by the shelter's staff is not known, but all men then referred participated in the study. Data saturation was reached with 18 individual interviews. Table 2 summarizes the demographic and chronic disease characteristics of study participants. Participants were aged between 32 and 65 years (mean = 48, Standard deviation = 8.52). Most participants (61.1%) had multiple chronic physical diseases, and 38.9% had a co‐morbid mental illness.

**TABLE 2:** Participants' background information

	Participants n = 18
Age (mean years, standard deviation)	48 (8.52)
Language used in interview	
English	13 (72.2)
French	5 (27.8)
Marital status (n, %)	
Single	13 (72.2)
Divorced	3 (16.7)
Separated	2 (11.1)
Country of origin	
Canada	16 (88.9)
Ethiopia	1 (5.6)
El Salvador	1 (5.6)
Mean no. of co‐morbidities	2.0
Chronic physical diseases (n, %)	
Multiple physical morbidities	11 (61.1)
Single physical morbidities	7 (38.9)
Chronic pain^a^	7 (26.0)
Chronic respiratory diseases	4 (14.8)
Diabetes	3 (11.1)
Chronic skin infection	2 (7.4)
Arthritis	2 (7.4)
Neurological conditions	2 (7.4)
Cardiovascular disease	2 (7.4)
Cancer	1 (3.7)
Hypercholesterolemia	1 (3.7)
Migraines	1 (3.7)
Carpel tunnel	1 (3.7)
Hearing impairment	1 (3.7)
Co‐morbid mental illness (n, %)	
Total	7 (38.9)
Depression	3 (16.7)
Other	1 (5.6)
Undisclosed	
Currently using drugs or alcohol (n, %)	
Yes	10 (55.6)
No	8 (44.4)
Highest level of education completed (n, %)	
Did not complete high school	3 (16.7)
High school	2 (11.1)
Post‐secondary diploma	10 (55.6)
Undergraduate university degree	3 (16.7)
Employed (n, %)	
Employed currently	6 (33.3)
Employed previously	12 (66.7)
Housing (n, %)	
Emergency overnight shelter	17 (94.4)
Community	1 (5.6)
Annual income (n, %)	
<$20 000	14 (77.8)
$20 000‐$39 999	3 (16.7)
Prefer not to say	1 (5.6)

^a^ Total exceed 18 because of multimorbidities.

### 3.2 Perceived importance of SM and SM Support

#### 3.2.1 SM important, despite competing demands to fulfil basic needs

**FIGURE 2:**
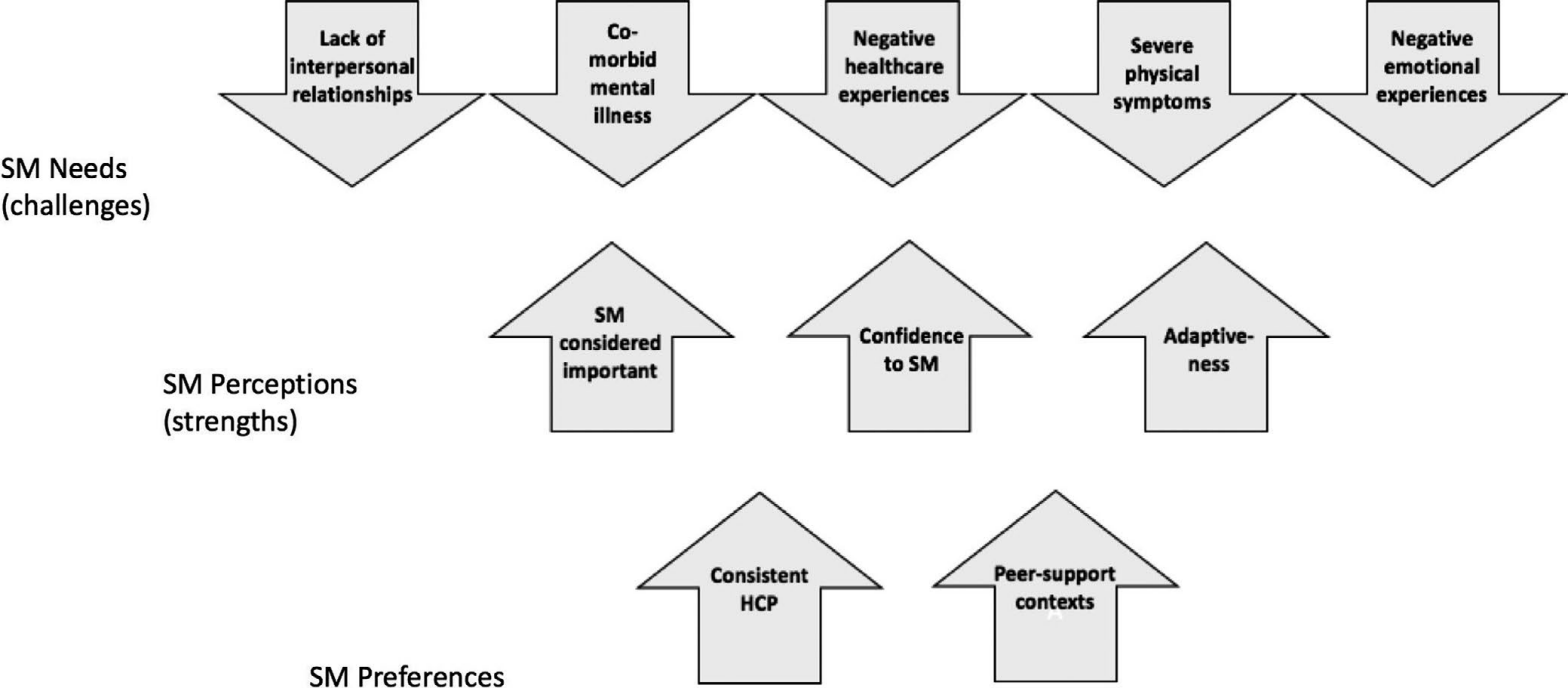
Self‐management preferences and perceptions of self‐management (strengths) are countered by SM needs (challenges)

Most participants (72.2%) agreed that chronic disease SM was important, despite the challenges to SM presented by homelessness (see Figure 2, perceptions). When comparing transcripts, no patterns were noted in the importance attributed to chronic disease SM according to disease, number of co‐morbidities, symptom severity or presence of co‐morbid mental illness. However, participants described daily life experiences that impinged on their ability to SM, including exposure to physical danger, harassment, theft, a lack of privacy, limited access to communication technologies, limited food choices, poor sleeping conditions, limited income and a lack of storage. As one participant said, ‘*It's not an easy life. People say that you're living for free, you're not … it's a lot of work* (participant 8)’. Several participants described having to choose between attending medical appointments and accessing a meal serving, or an employment opportunity.

#### 3.2.2 Participants confident to carry out and adapt SM behaviours

Almost all participants (88.9%) expressed confidence to carry out medical SM behaviours and described performing a number of SM behaviours (described in Table 3). Participants gained confidence by increasing their chronic disease knowledge and learning ‘*what it takes to get [chronic diseases] under control and manage them well* (participant 7)’. Participants described acquiring this knowledge mainly through trial and error, but also through the internet, books and directly from their HCPs.

**TABLE 3:** Participant self‐management behaviours (medical)

Medical SM behaviours
Modifying physical activity
Increasing physical activity (eg going to the gym)
Decreasing physical activity (eg stopping to rest during the day)
Modifying diet
Avoiding unhealthy foods (eg abstaining from sugar)
Seeking healthy foods (eg volunteering in exchange for vegetarian meal)
Navigating HCP relationship
Seeking information about disease (eg asking on‐site nurse about disease management)
Seeking medical support (eg seeing on‐site nurse or going to emergency room)
Advocating for better care (eg asserting right to information about medical procedure)
Sharing information with provider (eg carrying wireless cardiac rhythm device)
Making decisions
Monitoring symptoms (eg monitoring for signs of recurrent inflammation)
Analysing symptoms in light of acquired knowledge (eg linking asthmatic chest pain to increase in smoking)
Weighing pros and cons (eg living with joint pain vs. post‐operative recovery)
Deciding on action (eg going to emergency room when symptoms understood as critical)
Taking medications (eg over‐the‐counter, prescribed and illicit medications)
Linking mental state to physical well‐being (eg managing anxiety to reduce physical pain)
Context adapted SM behaviours
Minimizing symptom impacts
Improving sleeping conditions (eg seeking out a particular mattress)
Reducing migraine pain (eg using earplugs to block out sound)
Reducing fatigue (eg drinking coffee in lieu of having a place to rest)
Saving money
Non‐pharmacological pain management (eg alleviating back pain with hot showers at shelter)
Finding cheap alternatives (eg buying discount hearing aids)
Avoiding theft (eg keeping medications on one's person at all times)
Remembering SM regimen (eg keeping medications in bulky container in pocket as a reminder)

Most participants had to become creative in adapting particularly their medical SM behaviours to their resource‐limited setting. For example, one participant described using facecloths from the shelter to protect an open wound in lieu of unaffordable bandages. Another participant kept his medications inside an artificial plastic egg in his pants pocket, which served the dual purpose of avoiding theft and helping him to remember to take his pills. Other participants employed similarly adaptive SM behaviours to save money, reduce risks of infection, and minimize disease symptoms like fatigue, muscle pain and headache (See Table 3).

### 3.3 SM support needs

#### 3.3.1 Emotional SM is most challenging

Despite participants attributing importance to SM and expressing confidence to perform and modify their SM behaviours, managing negative emotions pertaining to the difficulties of chronic disease while homeless emerged as the aspect of SM that was most challenging (see Figure 2, SM needs). Many participants (61.1%) described feeling regularly challenged by their negative emotions, which included despair, frustration, guilt, regret, powerlessness, persecution, anger, fear, uncertainty and lack of motivation. Emotions around chronic disease and homelessness were intertwined; participants were generally unable to speak of their emotions pertaining to their chronic disease without describing how homelessness exacerbated their emotional challenges. For example, one participant described how the poor sleeping conditions at the shelter exacerbated his medication‐related fatigue and left him feeling frustrated:

I walked in [to the shelter] and guys were snoring … I can't sleep, so I gotta take [a sleeping pill] … or a drink, something to put yourself to sleep … then you wake up the next day and you're drowsy for the whole day … you never get out of the cycle (participant 9).

**FIGURE 3:**
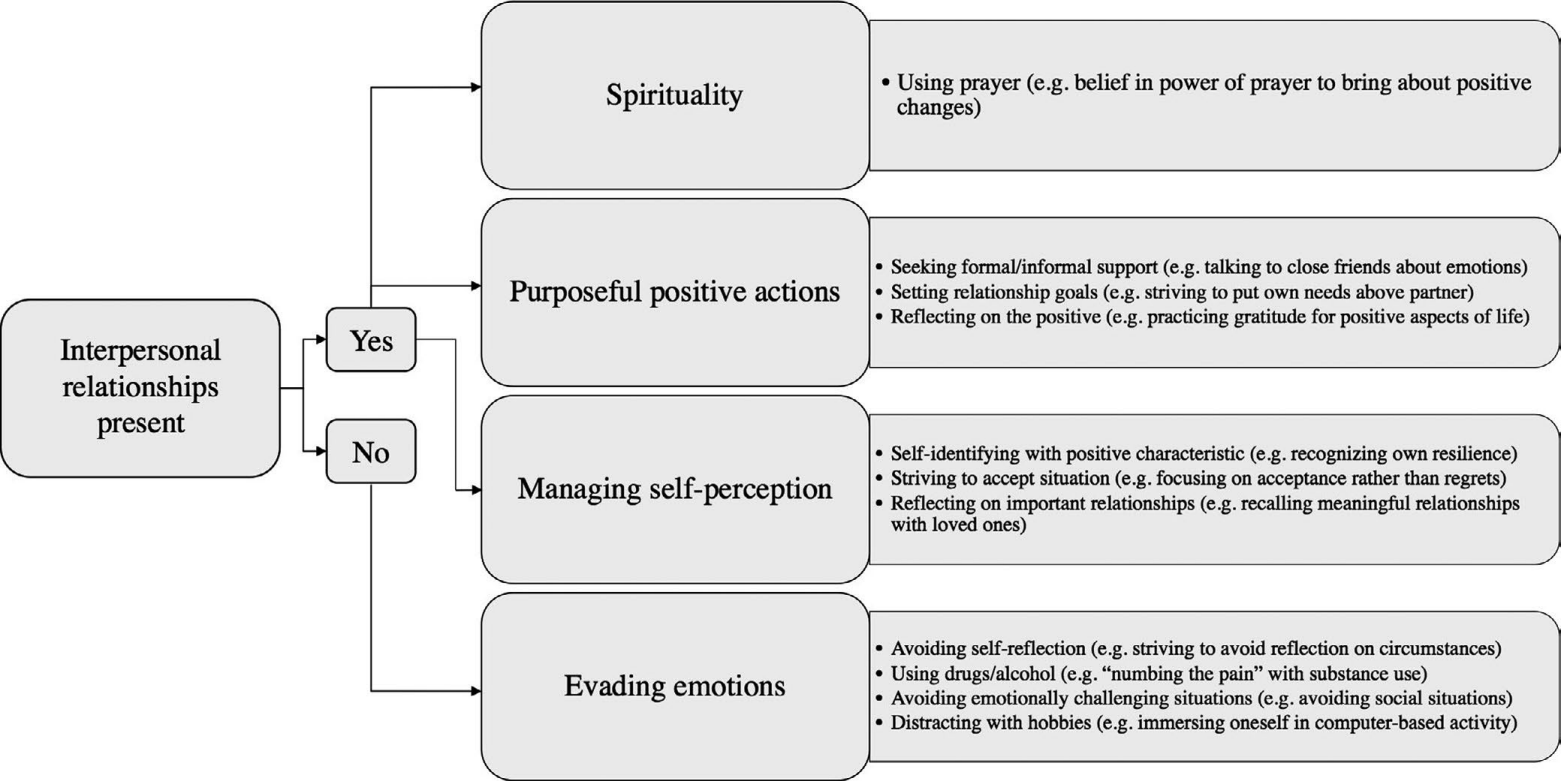
Type of emotional SM behaviours utilized tied to presence or absence of interpersonal relationships

Four categories of emotional SM behaviours were described by participants (See Figure 3): purposeful positive actions, managing self‐perceptions, spirituality and evading emotions. Participants generally using more than one. One of the most frequent emotional SM behaviours was purposeful positive actions, which includes actively seeking emotional support, taking stock of the positive things in their lives and/or setting goals about how to improve their emotional well‐being. Participants also utilized emotional SM behaviours that were labelled managing self‐perception to characterize their reflections on personal strengths or abilities, recalling their importance to loved ones or trying to accept their situations. Although described by fewer participants, strategies such as prayer, spiritual beliefs or seeking spiritual knowledge were labelled spirituality. Some participants also spoke of evading emotions through the use of drugs or alcohol or engaging in distracting activities.

#### 3.3.2 Emotional SM behaviours tied to presence of interpersonal relationships

When comparing transcripts, differences in emotional SM emerged based on the presence or absence of interpersonal relationships (see Figure 3). Most participants (66.7%) described having one or more on‐going interpersonal relationship with a friend or family member. These participants described utilizing three of the four aforementioned emotional SM behaviours: managing self‐perception, purposeful positive actions and spirituality, which overall sought to balance their negative emotional experiences with positive actions or thoughts. For example, one participant described ‘*Things are going to get better, so … you take it day by day … [I always try to] get myself into a better train of thought … Don't let it get you* (participant 7)’.

Conversely, those participants with no important relationships (33.3%) described predominantly using SM behaviours labelled as evading emotions in Figure 3, as one participant put it, ‘*fix the emptiness* (participant 9)’. These participants' descriptions of emotional SM was characterized by persistent and overwhelming negative emotions. One participant said ‘*[living on the street is like] … living in, in Iraq’* because ‘…*you're so used to being in pain or tired and frustrated …* (participant 9)’ Participants in this subgroup agreed that their social isolation contributed to their emotional challenges.

#### 3.3.3 SM needs high for participants with severe physical symptoms and co‐morbid mental illness

Two other subgroups of participants emerged as having high SM needs (see Figure 2, SM needs). First, participants with ‘severe’ physical disease symptoms (44.4%) described using almost twice as many medical SM behaviours as those with ‘non‐severe’ symptoms. Physical symptoms were coded as ‘severe’, if participants experienced disruptions to daily life, on‐going physical pain or discomfort, changes in cognition or frequent hospitalizations as a result of their chronic disease.

Second, participants with co‐morbid mental illness described managing additional emotional and cognitive symptoms related to their co‐morbid mental illness or treatments, including hopelessness, numbness, severe anxiety, lack of motivation, impulsivity, difficulty planning, fatigue, and sexual dysfunction. Participants in this subgroup managed these symptoms by utilizing both emotional SM and additional medical SM behaviours, including seeking education about their mental illnesses, taking psychiatric medications and monitoring medication effectiveness.

For some, mental illness compounded their day‐to‐day challenges by limiting their access to employment and contributing to their overall social isolation. As one participant explained, ‘*What life could you possibly have if you have anxiety attacks … like … you can't talk to anybody, you can't socialize with anybody* (participant 9)’.

Participants also described mental illness as adding confusion to the process of chronic disease SM. For example, two participants had difficulty determining whether their somatic symptoms were mental or physical in origin. This uncertainty made it more difficult to carry out appropriate SM behaviours.

### 3.4 Preferences for SM support

#### 3.4.1 Preferences for consistent, supportive HCP

Participants expressed a preference for consistent HCPs and peer‐support SM (see Figure 2, preferences). Participants with consistent relationships with a single HCP (33.3%) described having positive experiences when seeking support for their chronic diseases. These participants explained that their HCP's knowledge of their personal and medical history and the ability to ‘*talk about problems and personal things* (participant 8)’ allowed for support that was tailored to participant's individual circumstances. For example, one participant described how, ‘*I listen to my doctor … He's my doctor, but he's a friend. And he knows me* (participant 10)’. Despite two participants expressing that their friend‐like relationships with their HCPs sometimes created communication challenges, both expressed a preference for personal‐style relationships with their HCPs.

Conversely, participants with no regular HCP (66.7%) utilized health‐care services only in emergency situations or if they deemed their disease conditions as serious. Forming and maintaining relationships with HCPs was more difficult for these participants due to barriers establishing contact with the system. These barriers included the annual expiration of provincial health insurance for individuals with no permanent address (16.7%), difficulty navigating the health‐care system (27.8%), fears of medical treatments (22.2%) and negative past experiences with the healthcare system (38.9%). Perhaps the most deterring negative experience described by 22.2% of participants was the perception of prejudice on the part of healthcare workers, which undermined their confidence in the healthcare system and HCPs in general. As one participant described,

They [doctors] size you up and they're like … this guy is a street guy … And if he's a street guy than he's here for drugs … they basically blow off everything you say (participant 9).

About a third of participants (33.3%) described utilizing the services of the shelter's on‐site nurse. All of these participants expressed satisfaction with this service and described the nurse as their first line of medical assistance in lieu of attempting to see a physician.

#### 3.4.2 Peer‐support interventions

Half‐way through the study, one participant reflected on his positive experiences with a peer‐support therapy programme. He described the peer‐support programme's many benefits:

…it's good to have people to talk to … even if you knew people were just around … it just made you feel, sort of, better (participant 8).

Eight of the ten subsequent participants agreed that peer‐support SM interventions would be helpful in managing the emotional challenges of chronic disease and homelessness because, unlike HCPs, peers have ‘*been through all of it* (participant 12)’. Some participants reflected on the utility of sharing their experiences and strategies that they learned through many years of managing their chronic illness in the context of homelessness.

## 4 DISCUSSION

Chronic disease SM is effective in mitigating the impacts of chronic disease and shows promise among populations experiencing homelessness.^20^, ^21^, ^22^ However, to date little research has been conducted to explore the experience of chronic disease SM among this population. This study explored the perceptions, needs, and preferences of SM and SM support of men experiencing homelessness.

Despite competing demands to meet basic needs, participants perceived chronic disease SM as important. Similarly, a study by Henwood et al^21^ among men experiencing homelessness found that good health was never ‘irrelevant’ among participants; rather, SM became difficult to prioritize when faced with the demands of survival. The importance placed on SM has implications for disease outcomes, such disease perception has been linked to treatment adherence,^34^ self‐efficacy,^35^ social functioning, well‐being, distress, disease state and survival.^36^ In the present study, participants expressed confidence, or self‐efficacy, to carry out medical SM behaviours and adapt these to the context of homelessness in an effort to maintain SM behaviours despite competing demands to fulfil basic needs. Whereas resourcefulness among populations experiencing homelessness has been peripherally acknowledged in the literature,^37^ it has not yet been explored as a strength upon which to develop SM support interventions.

The greatest SM challenges participants described pertained to their emotions around homelessness and chronic disease. Whereas the prevalence of poor emotional well‐being among populations experiencing homelessness has received some attention in the literature,^38^, ^39^ the emotional intersection of chronic disease and homelessness has been largely unexplored. Participants' descriptions of emotional SM behaviours were consistent with active styles of emotional coping^40^ as well as with avoidant styles of emotional coping.^41^ Whereas active coping strategies tend to be associated with more favourable health and psychological outcomes, avoidant coping styles tend to be associated with poorer physical and psychological outcomes and may actually impede active coping.^40^, ^41^, ^42^ In comparing the transcripts, it was noted that those participants with no social support seemed to utilize more avoidant emotional coping styles. Numerous studies have identified a lack of social support as a contributor to vulnerability in both physical disease processes and psychological well‐being (See Cal, Ribeiro de Sá, Glustak, & Santiago, for a review).^43^ Conversely, the presence of social support has been associated with increased SM behaviours, positive disease outcomes^44^ and, among populations experiencing homelessness, enhanced well‐being^45^ and positive coping.^46^

In addition to participants with no social support, two additional vulnerable subgroup of participants were identified: those with severe physical symptoms (or those experiencing increased symptom burden) and those with co‐morbid mental illness. Increased symptom burden has been associated with lower health‐related quality of life, increased hospitalizations and medical costs, and decreased survival and functional status.^47^ A comprehensive review by Druss and Walker^48^ found that co‐occurrence of mental illness and physical disease is associated with elevated symptom burden, functional impairment, decreased length and quality of life, increased costs, and a two‐ to fourfold risk of premature mortality. Echoing the assertions of some participants in the current study, the review found that mental illness and related treatments can impede chronic disease SM and lead to a worsening of physical or mental symptoms.

Reflecting participants' preferences for personalized SM support from HCPs, a scoping review of patient experiences across 171 studies found that patients value healthcare that accounts for their individual circumstances and experiences.^49^ However, populations experiencing homelessness are less likely to have consistent primary care providers compared with housed individuals.^50^ Participants who lacked a consistent HCP experienced barriers to accessing services that were described in a recent study by Loignon et al^51^ The study found that both healthcare system complexity and stigma resulting from ‘social distance’ between HCPs and clients are key impediments to accessing care among low‐income populations (p. 5). Such negative health‐care experiences may contribute to the overutilization of emergency services by this population.^5^

Peer support was strongly endorsed among participants in this study. Whereas none of the SM studies among homeless populations utilized a peer‐support intervention, the study by Henwood et al^21^ generated a peer‐support environment through their participatory research approach, which contributed to the intervention's success. Furthermore, peer‐based SM interventions have been associated with increases in self‐efficacy, reduced pain, fewer emergency room visits,^52^ improvements in physical activity, greater smoking cessation,^53^ increased satisfaction with medical care, increased social support, better mood and increased sense of belonging.^54^ When taken with existing evidence of the importance of peer‐support and social relationships on psychological and physical well‐being^44^, ^45^, ^46^, ^53^ peer‐support and social network‐building SM interventions emerge as crucial strategies in developing SM behaviours among men experiencing homelessness.

### 4.1 Study limitations

As with much research on SM, self‐selection bias may leave out the most marginalized segments of this already hard‐to‐reach population.^55^ There is also a risk of social desirability bias.^56^ The study additionally failed to elicit sociodemographic data pertaining to individual's racialized identities, which may have implications for the experiences of prejudice described by some participants.

### 4.2 Practice implications

Barriers to developing chronic disease SM behaviours and SM support must be addressed at various levels of the healthcare system. At the governmental‐level, coordination and consistent funding could improve access to SM resources, such as on‐site health‐care and social services. Among service organizations, eliciting input from service users on policy development may reduce barriers, like access to personal storage, meal options or exercise spaces. Providing on‐site health‐care services outside of work hours may diminish competing demands between SM and employment. Increasing awareness and support of nursing services may foster linkages between this population and the health‐care system. At the level of HCPs and researchers, eradicating stigma and prejudice is paramount. The most vulnerable segments of this population require SM support interventions that address and support their complex medical and emotional needs, while simultaneously drawing upon their strengths.

### 4.3 Conclusion

The importance placed on chronic disease SM by study participants, their confidence to SM and their resourceful adaptation of SM behaviours are reflective of the many strengths and resiliencies upon which SM support interventions may be built. However, the intersecting challenges of chronic disease, homelessness, social isolation, severe physical symptoms and co‐morbid mental illness render some subgroup of this population particularly vulnerable to poor health and psychological outcomes. Whereas there is no substitute for SM support provided by a consistent and trusted HCP, participant's enthusiasm for peer‐support SM interventions provides a promising start.

## CONFLICT OF INTEREST

The authors whose names are listed immediately below certify that they have NO affiliations with or involvement in any organization or entity with any financial interest (such as honoraria; educational grants; participation in speakers' bureaus; membership, employment, consultancies, stock ownership, or other equity interest; and expert testimony or patent‐licensing arrangements), or non‐financial interest (such as personal or professional relationships, affiliations, knowledge or beliefs) in the subject matter or materials discussed in this manuscript. Author names: Laura Merdsoy, Sylvie Lambert and Jessica Sherman.

## ETHICAL APPROVAL

Ethics approval for the study was obtained from the McGill University Faculty of Medicine Institutional Review Board and the Chief Executive Officer of Welcome Hall Mission.

## Data Availability

Due to the sensitive nature of the qualitative data collected in this manuscript, research data are not shared.

## References

[1] World Health Organization . Preventing Chronic Diseases: A Vital Investment. WHO global report. Geneva: The Author; 2005 https://www.who.int/chp/chronic_disease_report/full_report.pdf. Accessed May 15, 2019.

[2] Canadian Nurses Association . Chronic disease and nursing: a summary of the issues CNA Backgrounder. 2005(October):1–7. https://cna‐aiic.ca/~/media/cna/page‐content/pdf‐en/bg3_chronic_disease_and_nursing_e.pdf. Accessed May 15, 2019.

[3] Public Health Agency of Canada , Centre for Chronic Disease Prevention and Control (Canada) . Improving Health Outcomes: A Paradigm Shift: Centre for Chronic Disease Prevention Strategic Plan 2016–2019. Ottawa, ON: Public Health Agency of Canada; 2015.

[4] Betancourt MT , Roberts KC , Bennett TL , Driscoll ER , Jayaraman G , Pelletier L . Monitoring chronic diseases in Canada: The Chronic Disease Indicator Framework. Chronic Dis Inj Can. 2014;34(1):1–30. 24898593

[5] Hwang SW , Aubry T , Palepu A , et al. The health and housing in transition study: a longitudinal study of the health of homeless and vulnerably housed adults in three Canadian cities. Int J Public Health. 2011;56(6):609–623. 21858461 10.1007/s00038-011-0283-3

[6] Lippert AM , Lee BA . Stress, coping, and mental health differences among homeless people. Sociol Inq. 2015;85(3):343–374.

[7] Pauly B . Close to the Street: Nursing Practice with People Marginalized by Homelessness and Substance Use In: Guirguis‐Younger M, McNeil R, Hwang SW, eds. Homelessness & Health in Canada. Ottawa, ON: University of Ottawa Press; 2014:211–232.

[8] Wagner EH , Austin BT , Davis C , Hindmarsh M , Schaefer J , Bonomi A . Improving chronic illness care: translating evidence into action. Health Aff (Millwood). 2001;20:64–78. 11816692 10.1377/hlthaff.20.6.64

[9] Adams K , Greiner AC , Corrigan JM . Report of a summit. The 1st annual crossing the quality chasm summit: A focus on communities. Washington, CD: National Academies Press; 2004. 25009886

[10] British Columbia Ministry of Health . Self‐management: a health care intervention. 2012 https://www.selfmanagementbc.ca/uploads/What%20is%20Self‐Management/PDF/Self‐Management%20Support%20A%20health%20care%20intervention%202011.pdf. Accessed June 10, 2011.

[11] Corbin J , Strauss A . Managing chronic illness at home: three lines of work. Qual Sociol. 1985;8(3):224–247.

[12] Lorig KR , Holman HR . Self management education: history, definition, outcomes and mechanisms. Ann Behav Med. 2003;26(1):1–7. 12867348 10.1207/S15324796ABM2601_01

[13] Curry SJ , Corrigan J . Priority areas for national action: transforming health care quality. Washington, DC: National Acadamies Press, Institute of Medicine; 2003. 25057643

[14] Jones MC , MacGillivray S , Kroll T , Zojoor AR , Connaghan J . A thematic analysis of the conceptualization of self‐care, self‐management, and self‐management support in the long‐term conditions management literature. J Nurs Healthc Chronic Illn. 2011;3(3):174–185.

[15] Holman H , Lorig K . Patient self‐management: a key to effectiveness and efficiency in care of chronic disease. Public Health Rep. 2004;119(3):239–243. 15158102 10.1016/j.phr.2004.04.002 PMC1497631

[16] Ryan P , Sawin KJ . The individual and family self‐management theory: background and perspectives on context, process, and outcomes. Nurs Outlook. 2009;57(4):217–225.e6. 19631064 10.1016/j.outlook.2008.10.004 PMC2908991

[17] Kidd T , Carey N , Mold F , et al. A systematic review of the effectiveness of self‐management interventions in people with multiple sclerosis at improving depression, anxiety and quality of life. PLoS One. 2017;12(10):e0185931. 29020113 10.1371/journal.pone.0185931 PMC5636105

[18] Dineen‐Griffin S , Garcia‐Cardenas V , Williams K , Benrimoj SI . Helping patients help themselves: a systematic review of self‐management support strategies in primary health care practice. PLoS One. 2019;14(8):e0220116. 31369582 10.1371/journal.pone.0220116 PMC6675068

[19] Pinnock H , Parke HL , Panagioti M , et al. Systematic meta‐review of supported self‐management for asthma: a healthcare perspective. BMC Med. 2017;15(1):64. 28302126 10.1186/s12916-017-0823-7 PMC5356253

[20] Hulton LJ , Strang S , Brooks S , Hostetter M . A pilot study of chronic disease self‐management in a homeless population. GSTF J Nurs Health Care. 2015;13(1):30.

[21] Henwood BF , Cabassa LJ , Craig CM , Padgett DK . Permanent supportive housing: addressing homelessness and health disparities? Am J Public Health. 2013;103(Suppl 2):S188–S192. 24148031 10.2105/AJPH.2013.301490 PMC3908899

[22] Savage C , Xu Y , Richmond MM , Corbin A , Falciglia M , Gillespie G . A pilot study: retention of adults experiencing homelessness and feasibility of a CDSM diabetes program. J Community Health Nurs. 2014;31(4):238–248. 25356993 10.1080/07370016.2014.958406

[23] Lorig KR , Sobel DS , Stewart AL , et al. Evidence suggesting that a chronic disease self‐management program can improve health status while reducing hospitalization: a randomized trial. Med Care. 1999;37(1):5–14. 10413387 10.1097/00005650-199901000-00003

[24] Chu J , Leino A . Advancement in the maturing science of cultural adaptations of evidence‐based interventions. J Consult Clin Psychol. 2017;85(1):45–57. 28045287 10.1037/ccp0000145

[25] Oxford English Dictionary. Perception. 2017.

[26] Chen J , Hou F . Unmet needs for health care. Health Rep. 2002;13(2):23–34. 12743954

[27] Doyal L , Gough I . The Theory of Human Need. New York, NY: Palgrave Macmillan; 1991.

[28] Ryan M , Farrar S . Using conjoint analysis to elicit preferences for health care. Br Med J (Clin Res Ed). 2000;320(7248):1530–1533. 10.1136/bmj.320.7248.1530 PMC1118112 10834905

[29] Sandelowski M . Whatever happened to qualitative description? Res Nurs Health. 2000;23(4):334–340. 10940958 10.1002/1098-240x(200008)23:4<334::aid-nur9>3.0.co;2-g

[30] Welcome Hall Mission . About. 2016; https://welcomehallmission.com/about‐us/. Accessed May 15, 2019.

[31] Boddy C . Sample size for qualitative research. Qualitative Market Research: An International Journal. 2016;19(4):426–432.

[32] Braun V , Clarke V . Using thematic analysis in psychology. Qual Res Psychol. 2006;3(2):77–101.

[33] Vaismoradi M , Turunen H , Bondas T . Content analysis and thematic analysis: Implications for conducting a qualitative descriptive study. Nurs Health Sci. 2013;15(3):398–405. 23480423 10.1111/nhs.12048

[34] Horne R , Weinman J . Self‐regulation and self‐management in asthma: exploring the role of illness perceptions and treatment beliefs in explaining non‐adherence to preventer medication. Psychol Health. 2009;17(1):17–32.

[35] Lau‐Walker M . Predicting self‐efficacy using illness perception components: a patient survey. Br J Health Psychol. 2006;11(Pt 4):643–661. 17032489 10.1348/135910705X72802

[36] Broadbent E , Wilkes C , Koschwanez H , Weinman J , Norton S , Petrie KJ . A systematic review and meta‐analysis of the Brief Illness Perception Questionnaire. Psychol Health. 2015;30(11):1361–1385. 26181764 10.1080/08870446.2015.1070851

[37] Dorsen C . Vulnerability in homeless adolescents: concept analysis. J Adv Nurs. 2010;66(12):2819–2827. 21073499 10.1111/j.1365-2648.2010.05375.x

[38] Biswas‐Diener R , Diener E . The subjective well‐being of the homeless, and lessons for happiness. Soc Indic Res. 2006;76(2):185–205.

[39] Coohey C , Easton SD . Distal stressors and depression among homeless men. Health Soc Work. 2016;41(2):111–119. 27263201 10.1093/hsw/hlw008 PMC4888093

[40] Carver CS , Scheier MF , Weintraub JK . Assessing coping strategies: a theoretically based approach. J Pers Soc Psychol. 1989;56(2):267–283. 2926629 10.1037//0022-3514.56.2.267

[41] Büssing A , Fischer J . Interpretation of illness in cancer survivors is associated with health‐related variables and adaptive coping styles. BMC Womens Health. 2009;9:2. 19178733 10.1186/1472-6874-9-2 PMC2661070

[42] Penley J , Tomaka J , Wiebe J . The association of coping to phsycial and psychological health outcomes: a meta‐analytic review. J Behav Med. 2002;25(6):551–603. 12462958 10.1023/a:1020641400589

[43] Cal SF , de Sa LR , Glustak ME , Santiago MB . Resilience in chronic diseases: A systematic review. Cogent Psychol. 2015;2(1).1024928.

[44] Strom JL , Egede LE . The impact of social support on outcomes in adult patients with type 2 diabetes: a systematic review. Curr Diabetes Rep. 2012;12(6):769–781. 10.1007/s11892-012-0317-0 PMC3490012 22949135

[45] Johnstone M , Jetten J , Dingle GA , Parsell C , Walter ZC . Enhancing well‐being of homeless individuals by building group memberships. J Community Appl Soc Psychol. 2016;26(5):421–438.

[46] Stein JA , Dixon EL , Nyamathi AM . Effects of psychosocial and situational variables on substance abuse among homeless adults. Psychol Addict Behav. 2008;22(3):410–416. 18778134 10.1037/0893-164X.22.3.410 PMC2806053

[47] Gapstur RL . Symptom burden: a concept analysis and implications for oncology nurses. Oncol Nurs Forum. 2007;34(3):673–680. 17573326 10.1188/07.ONF.673-680

[48] Druss BG , Walker ER . Mental disorders and medical comorbidity. Synth Proj Res Synth Rep. 2011;21:1–26. 21675009

[49] The Guidance Development Group , National Clinical Guideline Centre, NICE . Patient Experience in Adult NHS Services: Improving the Experience of Care for People Using Adult NHS services. Clinical Guidelines. London, UK: National Institute for Health and Care Excellence; 2012 https://www.nice.org.uk/guidance/cg138/resources/patient‐experience‐in‐adult‐nhs‐services‐improving‐the‐experience‐of‐care‐for‐people‐using‐adult‐nhs‐services‐pdf‐35109517087429. Accessed June 13, 2019.

[50] Khandor E , Mason K , Chambers C , Rossiter K , Cowan L , Hwang SW . Access to primary health care among homeless adults in Toronto, Canada: results from the Street Health survey. Open Med. 2011;5(2):e94–e103. 21915240 PMC3148004

[51] Loignon C , Hudon C , Goulet E , et al. Perceived barriers to healthcare for persons living in poverty in Quebec, Canada: the EQUIhealThY project. Int J Equity Health. 2015;14:4. 25596816 10.1186/s12939-015-0135-5 PMC4300157

[52] Parry M , Watt‐Watson J . Peer support intervention trials for individuals with heart disease: a systematic review. Eur J Cardiovasc Nurs. 2010;9(1):57–67. 19926339 10.1016/j.ejcnurse.2009.10.002

[53] Stanford School of Medicine . Chronic Disease Self‐Management Program (Better Choices, Better Health® Workshop). 2017 http://patienteducation.stanford.edu/programs/cdsmp.html. Accessed May 15, 2019.

[54] Hoey LM , Ieropoli SC , White VM , Jefford M . Systematic review of peer‐support programs for people with cancer. Patient Educ Couns. 2008;70(3):315–337. 18191527 10.1016/j.pec.2007.11.016

[55] Foster M , Kendall E , Dickson P , Chaboyer W , Hunter B , Gee T . Participation and chronic disease self‐management: are we risking inequitable resource allocation? Aust J Prim Health. 2003;9(3):132–140.

[56] Grimm P . Social desirabililty bias. Part 2. Marketing research Wiley International Encyclopedia of Marketing. 2010 10.1002/9781444316568.wiem02057. Accessed June 13, 2019.

